# Prediction of Genomic Islands in Three Bacterial Pathogens of Pneumonia

**DOI:** 10.3390/ijms13033134

**Published:** 2012-03-07

**Authors:** Feng-Biao Guo, Wen Wei

**Affiliations:** Center of Bioinformatics and Key Laboratory for NeuroInformation of Ministry of Education, School of Life Science and Technology, University of Electronic Science and Technology of China, Chengdu 610054, China; E-Mail: doramosquito@163.com

**Keywords:** genomic islands, pneumonic pathogens, cumulative GC profile

## Abstract

Pneumonia is one kind of common infectious disease, which is usually caused by bacteria, viruses, or fungi. In this paper, we predicted genomic islands in three bacterial pathogens of pneumonia. They are *Chlamydophila pneumoniae*, *Mycoplasma pneumoniae* and *Streptococcus pneumoniae*, respectively. For each pathogen, one clinical strain is involved. After implementing the cumulative GC profile combined with *h* and BCN index, eight genomic islands are found in three pathogens. Among them, six genomic islands are found to have mobility elements, which constitute a kind of conserved character of genomic islands, and this introduces the possibility that they are genuine genomic islands. The present results show that the cumulative GC profile when combined with *h* and BCN indexes is a good method for predicting genomic islands in bacteria and it has lower false positive rate than the SIGI method. Specially, three genomic islands are found to contain clusters of genes coding for production of virulence factors and this is useful for research into the pathogenicity of these pathogens and helpful for the treatment of diseases caused by them.

## 1. Introduction

Horizontal gene transfer is a process in which an organism incorporates genetic material from another organism without being the offspring of that organism. It is one of the most important ways for species to evolve, particularly microorganisms [1,2]. In special cases, large exogenous gene fragments (10–200 kb) in bacterial genomes constitute genomic islands. The alien genomic islands often contain genes associated with the survival of the organism under adverse conditions. According to the functions of genomic islands, they can be classified as pathogenicity islands, symbiosis islands, metabolic islands, secretion islands and resistance islands [3,4]. Among them, pathogenicity islands contain clusters of genes encoding virulence factors such as overt toxins, adherence factors, secretion proteins and molecules required for entry into the host cell or for acquisition of limiting metabolites [5].

Since the discovery of pathogenic islands in *Escherichia coli* [6], intensive studies have been performed on the identification of genomic islands in bacterial species. All of these works recur to the specific characters of horizontal gene transfer with respect to the host genome. These distinct characteristics include G + C content, codon usage, amino acid usage, dinucleotide usage and tetranucleotide relative abundance values *etc*. [7–9]. Among them, assessing the change of G + C content is an established way to detect the events of horizontal gene transfer. Traditionally, the sliding-window method is used to calculate the distribution of G + C content along a bacterial chromosome [7]. Although the window-based method is extensively used, the proper window size is hard to adjust. That is to say, small window size often leads to large statistical fluctuations, whereas large window size may bring about low resolution. To avoid the defections of sliding-window method, Zhang and Zhang proposed the cumulative GC profile [10], a windowless method, for computing the G + C content of DNA. This method has been successfully used to identify genomic islands in genomes of *Bacillus cereus* [11,12], *Corynebacterium efficiens* [13], *Corynebacterium glutamicum* [14], *Rhodopseudomonas palustris* [15], *Vibrio vulnificus* [14] and six plant pathogens [16]. In this paper, the cumulative GC profile method, combined with *h* index and biased codon number (BCN) has been used to detect genomic islands in three pneumonic pathogens.

## 2. Results

**Figure 1:**
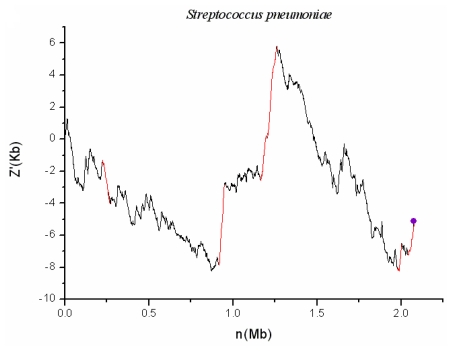
The z′ curve (or cumulative GC profiles) for genome of *S. pneumoniae* G54. All the identified genomic islands are denoted by red lines and adjacent RNA sites are marked by violet points.

In order to identify the genomic islands in three pneumonic pathogens, we combine cumulative GC profile (or named *Z_n_*′ curve) with two indices: *h* value and the BCN (biased codon number). The homogeneity of external nucleotide composition of genomic islands leads to the approximately linear section in the curve. The heterogeneity between foreign gene fragment and the native genome leads to sudden turning points beside the linear section. An increase in the cumulative GC profile or curve means a decrease in G + C content and vice versa. The *h* values are calculated for each linear section in all the three genomes. Next, if this linear section is not a ribosomal protein region and satisfies the conditions of *h* less than 0.1, and BCN exceeding 17 (*P* < 0.05), it will be identified as a genomic island. Usually, ribosomal proteins tend to adopt “optimal” codons and so have different codon usages with common genes. It is widely believed that the major cause for selection on codon usage of ribosomal proteins is that certain “optimal” codons are translated more accurately and/or efficiently. In contrast, laterally transferred genes tend to use odd codons owing to the different nucleotide composition between their host and donor. Generally speaking, with the exception of laterally transferred genes and highly expressed genes including ribosomal protein coding genes, all genes tend to have similar compositions in a certain genome. The curve for the *S. pneumoniae* G54 is illustrated in Figure 1 and the red lines denotes the identified genomic islands. Consequently, seven genomic islands are detected in *S. pneumoniae* G54 and one genomic island is found in *C. pneumoniae* CWL029. However, no one is found in *M. pneumoniae* M129. Details of these identified genomic islands are listed in Table 1.

### 2.1. *S. pneumoniae* G54

*S. pneumoniae* is a gram-positive organism and is the most common pathogen of pneumonia and meningitis. There are seven genomic islands detected in the chromosome, which correspond to red lines in Figure 1. The tRNA genes have been discovered to lie in the flanking regions of some genomic islands [6]. Indeed, SPGGI07 is found to have Arg-tRNA junction (3′), as shown in Figure 1. Owing to the heterogeneous of external genes, seven genomic islands have distinct codon usage with the native genome. After *t*-test at 5% significance level, 21, 20, 33, 21, 37, 31 and 21 biased codons are found to exist in the seven genomic islands, respectively. Integrases and/or transposases have been found to lie in the regions of six genomic islands, with SPGGI06 as an exception. In addition, SPGGI04, SPGGI05 and SPGGI07 have ISs (Insertion sequences) or transposons, whereas the others do not. Although SPGGI06 has not any integrase or transposase, both z′ curve plot and BLAST analyses show that this fragment is lost in most strains of *S. pneumoniae* and the other sequenced *Streptococcus* species. This could be taken as further evidence that SPGGI06 has horizontal origin. Finally, SPGGI02 and SPGGI03 contain a phage-associated gene (SPG_0281 and SPG_0963), respectively.

**Figure 2:**
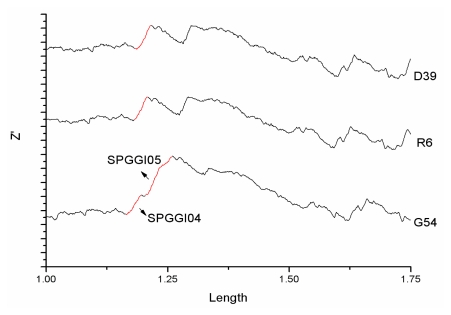
The z′ curves for genomes of three *S. pneumoniae* strains. For convenient observation, only part (1.0–1.75 Mb) of the chromosomes is drawn in the plot. Red lines denote genomic islands. As can be seen, SPGGI04-like fragments also exist in the two *S. pneumoniae* strains (D39 and R6). However, SPGGI05-like fragments disappear in them.

Actually, SPGGI04 and SPGGI05 are two connected parts of a larger genomic island located in 1164–1258 kb, which contains 100 genes. The fact that 43 conjugative transposons, 13 transposase-coding genes and an integrase-coding gene are contained in this larger island confirms the conclusion that it is an inserted fragment by horizontal transfer. As shown in Figure 2, SPGGI04-like fragments also exist in the other two strains (D39 and R6) of *S. pneumoniae*. However, SPGGI05-like fragments disappear in the two strains. Therefore, the larger GI should be divided into two parts (one is SPGGI04, the other is SPGGI05) in *S. pneumoniae* G54 genome. Both z′ curve and BLAST analysis show that SPGGI04 appears in the other 8 strains of *S. pneumoniae* but SPGGI05 appears only in 4 strians of *S. pneumoniae*.

Furthermore, a protein of iron acquisition system and a serine protease are contained in SPGGI03 and SPGGI07, respectively. This suggests they may be pathogenicity islands. The serine protease is associated with the ability of the pneumococcus to grow at high temperatures, to resist oxidative stress, and to undergo genetic transformation [17]. We have counted the integrases and transposase in the whole genome of *S. pneumoniae* G54. In the regions of genomic islands, the total number of such mobility elements is 19 and about 8% of genes belong to such elements. In contrast, there are a total of 88 integrases or transposases in the other region. On average, about 4% of genes in the retaining regions belong to such mobility elements. In addition, 20% and 4% of genes in the genomic islands and the other regions locates around another type of mobility element, *i.e.*, IS (insertion sequence) or transposon. Therefore, genes in the genomic islands have the much higher probability to locate near mobility elements when compared with the other regions.

### 2.2. *C. pneumoniae* CWL029

**Figure 3:**
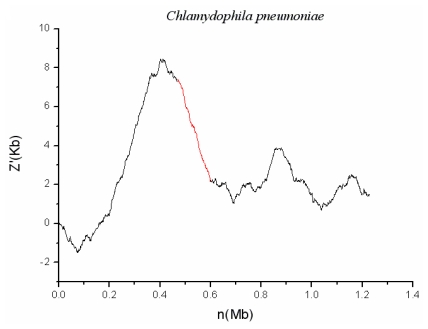
The z′ curve (or cumulative GC profiles) for genome of *C. pneumoniae* CWL029. The identified genomic island is denoted by red line.

*C. pneumoniae* is a kind of gram-negative obligate intracellular bacterium that has two distinct morphological forms. When outside the host cell, it exhibits small (0.3 μm) and just elementary body (EB). Whereas it turns into large (1.0 μm) and replicating form, which is called reticulate body (RB), when living intracellular. The z′ curve for *C. pneumoniae* is shown in Figure 3 and suggests a GI named CPnGI01 in the chromosome. This predicted genomic island contains 89 genes, one of which encodes hemolysin protein. Hemolysin is a kind of toxin that causes lysis of red blood cells *in vivo* [18]. Therefore, this island may be a pathogenicity island. In addition, significant codon usage bias also has been found between CPnGI01 and the host genome. After *t*-test at 5% significance level, 18 biased codons, are found to exist in CPnGI01. It would be complete if this fragment contained some mobility elements (such as integrases, transposases, IS elements, transposons and so on). Although CPnGI01 has not any mobility elements, both z′ curve plots and BLAST analysis show that this fragment appears only in the species *C. pneumoniae* and not in the other species of the genus *Chlamydophila*. This confirms that CPnGI01 has alien origin.

### 2.3. *M. pneumoniae* M129

**Figure 4:**
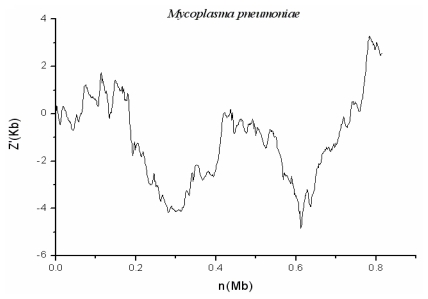
The z′ curve (or cumulative GC profiles) for genome of *M. pneumoniae* M129. There was not any identified genomic island.

*M. pneumoniae* is intermediate in size between typical bacteria and viruses. It has no cell wall and is unidentifiable in gram stains of sputum samples. It transmits through respiratory droplet and could damage respiratory epithelium of human beings, no matter whether adult or not, from the trachea to bronchioles. As shown in Figure 4, no genomic islands in *M. pneumoniae* genome are detected by using the systematic method.

## 3. Discussion

### 3.1. Conserved Features of Identified Genomic Islands

Genomic islands often have relatively less range of the GC content variation, compared with that of the whole genome [12–16]. Because of the above reason, the curve corresponding to a genomic island is always an almost straight line. It is not unexpected that values of the index, h, which measures the relative homogeneity of genomic islands to the whole genome, for the eight detected genomic islands are very small (max.: 0.083, min.: 0.011). Distinct codon usage is another feature of genomic islands. Base on *t*-test at significance level of 0.05, all identified genomic islands are found to have 18 or more biased codons, when compared with the host genomes. Mobility elements are also one of conserved characters of genomic islands. Here, six out of eight genomic islands are found to have mobility elements.

Recently, Vernikos and Parkhill [19] evaluated the frequently mentioned eight structureal features of genomic islands and ranked the relative importance of them by a machine learning approach. According to their results, composition bias and size are the most informative ones among those conserved features, followed by integrase or integrase-like protein domains. In this paper, all of the eight detected genomic islands meet the first important feature, according to BCN index. Furthermore, the size of all of the eight islands is between 12 kb and 54 kb. This means that all of them satisfy the first two important features. For the feature of integrase or transposase, 6/8 = 75% of the identified islands comes up to the standard. Most of the genomic islands detected in this work meet these frequently mentioned conserved features.

It is noteworthy that three of eight genomic islands contain pathogenicity-related encoding genes. This means that they may be pathogenicity islands. Identification of these islands will be of benefit to the research of pathogenicity for related pathogens and helpful for the treatment of diseases caused by them.

### 3.2. Comparison of the Cumulative GC Profile with SIGI Method

Waack *et al*. [20] developed a score-based method, named SIGI, for identifying horizontally transferred genes and genomic islands. Langille *et al*. [7,21] evaluated six existing genomic-islandpredictors using a comparative genomics approach. Among the six methods, they found that SIGI performed the best with 92% precision (though only 33% recall). That is to say, SIGI has the lowest false positive prediction. However, the true positive prediction of it is also quite low. Prediction results of SIGI for hundreds of bacterial genomes are available at IslandViewer website [21]. According to the data, 15 genomic islands are detected by SIGI method in the three pneumonic pathogens. After comparison, 6 of the 15 genomic islands overlap with SPGGI01 (containing 1 SIGI genomic island), SPGGI03 (containing 2 SIGI genomic islands), SPGGI04 (containing 2 SIGI genomic islands) and SPGGI05 (containing 1 SIGI genomic island). That is to say, overlapping predictions appear only in genome of *S. pneumoniae* G54 and not in the other two pathogens. Furthermore, 2 of the 9 (2/9 = 22.2%) uniquely predicted SIGI genomic islands possess mobility elements. In our results, 2 of the 4 (2/4 = 50%) uniquely predicted genomic islands have such elements. As widely accepted, mobility element is one of the important and highly conserved features of genomic islands. Therefore, the above analysis suggests that the two methods are very different and the cumulative GC profile may have a lower false positive rate than the SIGI method. Different results of the two methods would derive from their different principles. The systematic method used in this work is basically one kind of graphical method, whereas SIGI rely on a complicated statistical model.

It is also noteworthy that most of genomic islands detected by the SIGI method usually have small sizes. For example, 10 among the 15 (10/15 = 66.7%) SIGI genomic islands predicted in the three pneumonic pathogens are shorter than 10 kb. Whereas the usual size of genomic islands are in the range between 10 kb and 200 kb. Therefore, it may be possible that the SIGI method is more applicable to discovering horizontally transferred genes, rather than genomic islands.

## 4. Experimental Section

### 4.1. Data Source

For the three pneumonic pathogens, we downloaded the complete DNA sequences and related annotation information from NCBI ftp site [22]. They are *Chlamydophila pneumoniae* CWL029 (NCBI accession: NC_000922), *Mycoplasma pneumoniae* M129 (NC_000912) and *Streptococcus pneumoniae* G54 (NC_011072).

### 4.2. Cumulative GC Profile for Demonstrating GC Content Deviation of DNA Sequence

The method of the cumulative GC profile proposed by Zhang and Zhang [14] has been used to identify genomic islands in dozens of prokaryotic genomes. To accurately identify genomic islands, this method should be combined with *h* index and one method used to measure codon usage bias. In this paper, the methods of cumulative GC profile and *h* index remain the same as those proposed by Zhang and Zhang [13,14]. The methods are described briefly as follows.

Define

(1) $$Z_{n}=(A_{n}+T_{n})-(C_{n}+G_{n}),n=0,1,2,\ldots ,N,Z_{n}\in [-N,N]$$

In the above equation, *A_n_*, *C_n_*, *G_n_* and *T_n_*, are the cumulative numbers of the bases *A*, *C*, *G*, and *T*, respectively, occurring in the subsequence from the first base to the *n*-th base in the inspected DNA sequence with length *N. Z_n_* is one of the components of the *Z* curve [23]. To amplify the deviations of *Z_n_*, the curve of *Z_n_*~*n* is fitted by a straight line by using the least-squares approach.

(2) Z=k×n

In the equation of 2, (*Z*, *n*) is the coordinate of a point on the straight line fitted and *k* is its slope. Instead of using the curve of *Z*_n_~*n*, we will use the *Z_n_*′ curve, or cumulative GC profile, hereafter, where

(3) $$Z_{n}^{\prime }=Z_{n}-Z=Z_{n}-k\times n$$

### 4.3. The *h* Index for Evaluating the Homogeneity of the GC Content of Genomic Island

Genomic islands usually have fairly homogeneous GC contents and this fact implies that *Z_n_*′ is approximately equal to 0 [12–16]. An index named *h*, which describes the homogeneity of GC content of genomic island, is defined by the following equation.

(4) $$h=\frac{d_{gi}}{d_{c}}=\sqrt{\frac{\sum_{n=1}^{M}{\left({Z^{\prime }}_{n}\right)}^{2}/M}{\sum_{n=1}^{N}{\left({Z^{\prime }}_{n}\right)}^{2}/N}}$$

where *M* and *N* are the lengths of genomic island and chromosome, respectively. Symbol d denotes the deviation of the GC content from a constant for a whole genome or a genomic island. The so defined *h* index measures the relative magnitude of the GC content variations in a genomic island compared with that of the whole genome. If *h* is far less than 1, the variations of GC content of genomic islands may be considered to be small. In this work, *h* = 0.1 is taken as the criterion for deciding a potential genomic island.

It has been found that genes near the replication terminal are relatively GC-poor in bacterial genomes [24,25]. In contrast, in some archaeal genomes genes situated near OriC are relatively GC-poor [26]. For avoiding false positive predictions of genomic islands produced by replication-associated mutational pressure, we checked replication terminal regions of the three bacterial genomes. Although they are indeed relatively GC-poor, the homogeneities of them are so much weaker that their *h* indices are all bigger than 0.1. Therefore, they could not be predicted as genomic islands by our method.

### 4.4. BCN as an Index to Evaluate Codon Usage Bias

Instead of cub curve in Zhang and Zhang’s original method, biased codon number (BCN) is used to evaluate codon usage biases between a genomic island and the rest genome. With this slight modification, this method is thoroughly window-less. Furthermore, the systematic method becomes to be more statistically rigorous because cub curve is only one graphic method without statistical support. Genomic islands and the rest of the host genome could be regarded as two groups of samples. In each group, hundreds or dozens of genes are contained. For each gene, we can calculate the frequencies of 62 or 61codons (excluding two stop codons for Mycoplasma or three stop codons for others). Using 2-tailed *t*-test, we could determine whether the frequency of a given codon is significantly different between the two groups or not. Finally, the number of all the significantly biased codons could be calculated. This number, named BCN is used to measure the different between the codon usage of genomic islands and the rest of the host genome. If BCN is larger than or equal to 17, based on *t*-test at the significance level of 0.05, and *h* is less than 0.1, the candidate fragment will be predicted as genomic island. It should be noted that this systematic method applies to predicting only genomic islands rather than individual horizontal transferred genes. Like the other methods based on compositional biases, the systematic method could not detect those genomic islands whose donor has similar nucleotide composition with the host.

## 5. Conclusions

By replacing cub curve with BCN, we slightly modified the systematic method originally proposed by Zhang and Zhang for predicting genomic islands in bacterial genomes. With the modified method, we found eight genomic islands in three bacterial pathogens of pneumonia. Among them, six genomic islands have mobility elements, which constitute a kind of conserved character of genomic islands, and this adds the possibility that they are genuine genomic islands. Comparative analysis shows that the cumulative GC profile when combined with *h* and BCN indexes is a good method for predicting genomic islands in bacteria and it has lower false positive rate than SIGI method. In particular, three genomic islands are found to contain clusters of genes coding for production of virulence factors and this is useful for research into the pathogenicity of these pathogens and helpful for the treatment of diseases caused by them.

## Figures and Tables

**Table 1:** Genomic islands identified in the two pneumonic pathogens *C. pneumoniae* and *S. pneumoniae*.

Species	Segment	Start	End	*h* value	BCN	T (Transposase) or I (Integrase)	IS or Transposon
*C. pneumoniae* CWL029	CPnGI01	470980	599631	0.034	18		
*S. pneumoniae* G54	SPGGI01	137637	150199	0.048	21	T (SPG_0139, SPG_0140)	
*S. pneumoniae* G54	SPGGI02	224334	271125	0.021	20	I (SPG_0281)	
*S. pneumoniae* G54	SPGGI03	918880	947610	0.040	33	T (SPG_0987)	
*S. pneumoniae* G54	SPGGI04	1164899	1194336	0.057	21	T (SPG_1195, SPG_1196, SPG_1200- SPG_1207)	Transposon (SPG_1225, SPG_1227, SPG_1228) IS (SPG_1196, SPG_1202-SPG_1207)
*S. pneumoniae* G54	SPGGI05	1205234	1258720	0.083	37	I (SPG_1258) T (SPG_1260)	Transposon (SPG_1242- SPG_1245, SPG_1250- SPG_1252, SPG_1259, SPG_1263-SPG_1267, SPG_1270-SPG_1273, SPG1275, SPG_1282, SPG_1285, SPG_1287- SPG_1293)
*S. pneumoniae* G54	SPGGI06	1988635	2002624	0.011	31		
*S. pneumoniae* G54	SPGGI07	2040970	2078937	0.077	21	T (SPG_2157- SPG_2159)	IS (SPG_2157-SPG_2159)
